# Integrated comprehensive care in short-stay psychiatric units: a yoga-based adjunctive intervention and its association with wellbeing and patient satisfaction

**DOI:** 10.3389/fpubh.2026.1772247

**Published:** 2026-03-11

**Authors:** Laura Tolbaños-Roche, Mª del Mar Sánchez Delgado, Esperanza Bosch Casañas, Elisa Martín Gamero, Zaira Raquel Sanabria Medina, Claudio-Alberto Rodríguez-Suárez

**Affiliations:** 1 Department of Clinical Psychology, Psychobiology and Methodology, Section of Psychology, Faculty of Health Sciences, University of La Laguna, San Cristóbal de La Laguna, Spain; 2 Psychiatric Service, Insular Maternal and Child University Hospital Complex (CHUIMI), The Canary Islands Health Service (SCS), Las Palmas, Canary Islands, Spain; 3 Research Support Unit, Insular Maternal and Child University Hospital Complex (CHUIMI), The Canary Islands Health Service (SCS), Las Palmas, Canary Islands, Spain; 4 Department of Nursing, Faculty of Health Sciences, University of Las Palmas de Gran Canaria (ULPGC), Las Palmas, Canary Islands, Spain

**Keywords:** comprehensive care, integrative health, mental health, short-stay psychiatry, treatment satisfaction, wellbeing, yoga therapy

## Abstract

**Introduction:**

The treatment of patients in Short-Stay Psychiatric Units (SSPUs) requires a holistic approach that promotes overall health, enhances patients’ personal resources, and supports adaptation to daily life, thereby contributing to a more humane inpatient experience. The role of mental health professionals must extend beyond a traditional symptom-focused approach, with the therapeutic relationship grounded in empathy to provide a safe and trusting environment that enables patients to enhance self-confidence and actively engage in recovery. Preliminary evidence suggests yoga may improve functional outcomes and hospitalization experience.

**Methods:**

This study examined the differential effect of a yoga intervention implemented as an adjunct to comprehensive patient care in the SSPU of the Insular Maternal and Child University Hospital Complex of Las Palmas de Gran Canaria, Spain. The sample comprised 100 patients (52 in the experimental group and 48 in the control group) who completed the Distress Thermometer, the EuroQoL EQ-5D-5L Health Questionnaire, the Client Satisfaction Questionnaire (CSQ-8), and an open-ended questionnaire assessing treatment satisfaction.

**Results:**

Statistically significant improvements were observed following the comprehensive intervention, including reductions in perceived stress and health and functional problems, as well as improvements in self-rated overall health. At the between-group level, the experimental group showed significantly greater improvement in the self-care dimension of the EuroQoL EQ-5D-5L and higher overall treatment satisfaction on the Client Satisfaction Questionnaire. Patients’ qualitative descriptions characterized yoga as a beneficial component of care, contributing to relaxation, emotional regulation, feelings of peace and safety, and improved communication and empathy. Co-occurrence analysis suggests that these experiences, together with body awareness, are closely interrelated, reflecting experiential patterns rather than distinct therapeutic mechanisms.

**Discussion:**

This study shows that integrating a yoga-based intervention into comprehensive inpatient care is feasible, well-received, and valued by patients, supporting its potential as a complementary therapeutic activity.

## Introduction

Mental health disorders constitute a major global public health challenge. According to the World Health Organization (WHO), over one billion people worldwide live with a mental health disorder, with anxiety and depression being the most prevalent conditions, particularly among women. Severe mental disorders, including schizophrenia and bipolar disorder, are of particular clinical concern, with acute schizophrenia among the most disabling conditions. Suicide remains a leading cause of death among young people worldwide (1).

In Spain, the prevalence of mental health problems has risen markedly over the past decade, with a sharp rise during the COVID-19 pandemic. This surge has worsened pre-existing conditions such as eating disorders, psychotic episodes, and addictive behaviors, particularly in vulnerable populations including individuals with prior health conditions, healthcare workers, socioeconomically disadvantaged groups, and women (2). Since 2020, suicide rates have increased, with men accounting for approximately 74% of cases (3). In the Canary Islands, an estimated 2.5% of the population lives with a severe mental disorder, and regional suicide rates from 2007 to 2019 exceeded the national average, though both remain within the WHO-defined “medium” range (4).

The growing demand for mental health services has increased consultations in specialized psychiatric care, while limited public resources have driven growth in the private sector, where nearly 80% of psychiatric consultations now occur. This situation creates a pattern of inverse care, in which individuals with fewer economic resources, who are also at higher risk for mental health problems, face reduced access to specialized services. To address these challenges, the Spanish Ministry of Health launched the Mental Health Strategy 2022–2026, promoting a human-rights-based, community-oriented model that emphasizes recovery, equity, social inclusion, and person-centered care (5).

Short-Stay Psychiatric Units (SSPUs) provide specialized inpatient care for individuals experiencing acute psychiatric episodes with severe symptom exacerbation and functional disruption. Patients admitted to SSPUs often present with heterogeneous diagnoses, including schizophrenia, bipolar disorder, personality disorders, and eating disorders. While pharmacological treatment is essential, it alone is insufficient to address the multidimensional nature of acute psychiatric crises. Accordingly, the SSPU at the Insular University Hospital of Gran Canaria implements a humanized, integrated care model that strengthens personal resources, promotes functional adaptation, and fosters recovery-oriented inpatient experiences.

This approach aligns with the salutogenic framework, which emphasizes the promotion of health and wellbeing by enhancing protective resources, fostering healthy behaviors, and strengthening social support, rather than focusing solely on pathology (6). For individuals with severe mental disorders, such as schizophrenia, a salutogenic perspective supports symptom management, coping, and the development of personal strengths, contributing to improved self-esteem, identity, and sense of coherence (7). Social support, a nurturing environment, and positive self-identity are key resilience-building factors in inpatient mental healthcare (8). Effective implementation requires interdisciplinary collaboration among psychiatrists, psychologists, nurses, social workers, and occupational therapists, as well as empathic, trust-based therapeutic relationships.

Yoga, one of the most widely practiced mind–body disciplines globally, engages over 500 million practitioners worldwide, with approximately 12% of the Spanish population participating regularly. Recognized by the United Nations for its holistic contribution to health, June 21 has been designated as the International Day of Yoga (9, 10). Evidence demonstrates yoga’s effectiveness in managing stress, promoting relaxation, and supporting emotional regulation (11, 12). While benefits for anxiety and depression are well established (13, 14), research on yoga’s effects in severe mental disorders, particularly schizophrenia, remains limited.

Existing studies suggest that yoga, as an adjunctive therapy, may improve general symptom severity, negative and depressive symptoms, functional outcomes, and quality of life (15–18), though short-term inpatient interventions remain underexplored. In contrast, more evidence exists for multimodal interventions delivered in day-hospital or brief outpatient settings. Nevertheless, several investigations have demonstrated meaningful improvements in both physical and psychological outcomes following comprehensive inpatient treatment for individuals with mental disorders. For example, Sadlonova et al. (19) reported that 160 patients in an integrated psychosomatic inpatient program, including individual and group psychotherapy, psychoeducation, art therapy, relaxation training, and body-oriented and physical therapies, including structured exercise, experienced significant short- and long-term reductions in psychological symptoms and physical complaints, as well as improvements in health-related quality of life.

Hospitalization in SSPUs can be stressful, disrupting daily routines, reducing autonomy, and sometimes involving coercive measures, which may exacerbate emotional distress and negatively affect patient satisfaction (20). Holistic, patient-centered interventions are associated with greater satisfaction, which predicts better adherence, engagement, and therapeutic outcomes (21, 22). Preliminary studies indicate that even brief yoga interventions can enhance mood and improve patients’ perceptions of their hospitalization experience (23, 24).

Within this context, the present study evaluates the integration of a structured yoga intervention as an adjunct to comprehensive care in an SSPU. The primary objective is to assess the intervention’s effect on patients’ wellbeing by comparing comprehensive care alone (control group) with comprehensive care plus yoga (experimental group). It is hypothesized that the experimental group will show greater reductions in emotional distress (Hypothesis 1) and demonstrate improvements in overall health and functional capacity (Hypothesis 2) compared to the control group. Secondary objectives include assessing patient satisfaction with treatment and examining group differences, with the expectation that patient satisfaction will be high overall and higher in the experimental group (Hypothesis 3). Finally, the study examines changes in wellbeing from admission to discharge across all patients, hypothesizing that the comprehensive inpatient care will reduce emotional distress and improve health and functional outcomes regardless of group assignment (Hypothesis 4).

## Methods

### Study design

This study employed a mixed-methods design comprising a randomized controlled trial (RCT) and a qualitative component. The RCT was conducted in accordance with the Consolidated Standards of Reporting Trials (CONSORT) guidelines. The qualitative component was conducted following the Standards for Reporting Qualitative Research (SRQR). Participants were recruited using a non-probabilistic consecutive sampling approach due to the clinical and organizational characteristics of admissions to the Short-Stay Psychiatric Unit (SSPU). Following recruitment and baseline assessment, participants were randomly allocated to either the experimental group or the control group using a stratified randomization procedure. Randomization was performed to ensure comparability between groups and to preserve internal validity. The experimental group received the comprehensive care approach supplemented by a specific yoga intervention, whereas the control group received the comprehensive care approach alone. Two assessments—pre-test (before the intervention) and post-test (after the intervention)—were administered for the dependent variables of distress, general health status, and patient functionality. Additionally, a single assessment was conducted at the end of the inpatient stay to evaluate the number of physical restraints applied and the level of satisfaction with the treatment received.

### Participants

Participants were individuals admitted to the SSPU between January 14 and June 12, 2025, with a clinically diagnosed mental disorder based on standardized diagnostic criteria (DSM-5-TR/ICD-10-CM), confirmed through assessment by qualified mental health professionals. The inclusion criteria were men and women aged 18–70 years, without severe physical disabilities and with no recent history of major surgery. Participants were excluded or withdrawn from the study if they presented severe physical limitations that prevent safe participation in yoga practice, such as severe mobility impairment (e.g., inability to independently transition between standing and floor positions), recent major surgery, unstable cardiovascular conditions, or neurological disorders affecting balance or movement, and other medical conditions that contraindicate physical exertion, declined or were unable to complete the assessment instruments, or attended fewer than two yoga sessions, thus failing to meet the minimum exposure required for inclusion in the analyses.

### Variables and instruments

The study included both categorical and numerical variables. Categorical variables comprised: gender, diagnostic group, and treatment allocation (control vs. experimental). Numerical variables included age and scores derived from the clinical assessment instruments used in the study. Descriptive statistics for categorical variables are presented as frequencies and percentages, while numerical variables are summarized using appropriate measures of central tendency and dispersion (means, standard deviations, medians, and interquartile ranges), depending on data distribution.

The administration of the standardized questionnaires used to measure the dependent variables was carried out by a psychologist to ensure appropriate administration conditions and proper understanding of the completion instructions.

Distress was measured using the Spanish adaptation of the Distress Thermometer (25), a single-item questionnaire on a visual analog scale, ranging from 0 to 10, originally developed by Roth et al. (26). It is a self-assessment tool for emotional distress, in which participants rate the level of distress experienced over the past week, from 0 (“no distress”) to 10 (“extreme distress”). Convergent validity of the Spanish version was established through ROC analysis, indicating a modest discriminatory capacity (Area Under the Curve = 0.631), with sensitivity of 93%, specificity of 76%, positive predictive value of 82%, and negative predictive value of 90%. Given that this AUC is only moderately above chance level (0.5), the Distress Thermometer is best regarded as a screening instrument rather than a diagnostic test (27), and was therefore used in the present study solely to assess perceived distress levels.

To assess general health status and patient functionality, the EuroQoL EQ-5D-5L Health Questionnaire was used. This instrument comprises five dimensions: mobility, self-care, usual activities, pain/discomfort, and anxiety/depression, each rated on a 5-point Likert scale (No problems, Slight problems, Moderate problems, Severe problems, and Extreme problems/Unable to perform). The questionnaire also includes a visual analog scale (VAS) from 0 to 100 to assess the participant’s current overall health status. The construct validity of this questionnaire has been confirmed, demonstrating it to be a valid instrument for measuring perceived health in the general population (28).

Patient satisfaction with the treatment received during admission and stay in the SSPU was assessed using the Spanish version of the Client Satisfaction Questionnaire, CSQ-8 (29), Spanish translation by Vázquez et al. (30). The CSQ-8 consists of 8 items rated on a 4-point Likert scale. Total scores range from 8 to 32, with higher scores indicating greater satisfaction with the received service. The internal consistency of the original Spanish version is satisfactory, with a Cronbach’s alpha of 0.80.

The recording of the number of physical restraints was performed by the nursing staff of the SSPU, using the Form 8,101–24-Hour Nursing Assessment in Mechanical Restraint.

Patients’ perceptions of the therapeutic activities were evaluated using a brief, open-ended questionnaire designed by the team of psychiatrists and psychologists involved in the study to gather qualitative insights regarding recovery and treatment experience. The questionnaire consisted of two main questions, each with a clarifying follow-up:

1. Do you think the activities carried out during your stay in the SSPU have contributed to your recovery?
  - Follow-up: If yes, in what way?
2. Which activity has been the most helpful to you during your stay?
  - Follow-up: Why?

Finally, a sociodemographic questionnaire was also used to collect data on diagnosis, comorbidity, family history, medication use, and lifestyle habits such as alcohol and/or drug use and physical activity.

### Procedure

The study was approved by the Clinical Research Ethics Committee of the University Hospital of Gran Canaria Dr. Negrín (Las Palmas).

After the patient’s admission and the signing of the informed consent form, for those who agreed to participate in the study, each patient was randomly assigned to either the control or experimental group using a stratified randomization method, taking into account age (≤40 and >40 years) and gender (female vs. male). Participant recruitment was conducted continuously over a period of 5 months.

After completing the Distress Thermomether and the EuroQoL EQ-5D-5L Health Questionnaire to obtain pre-test measurements of the dependent variables, patients assigned to the control group received the comprehensive psychotherapeutic treatment, while those in the experimental group underwent the same treatment and, in addition, attended two weekly, one-hour yoga sessions until hospital discharge. The average length of stay in the SSPU is 3 weeks. A minimum attendance of two yoga sessions was established. Participants who attended fewer than two sessions were excluded from the overall data analysis.

During hospitalization, the nursing staff completed the Physical Restraints Questionnaire for each patient.

Prior to discharge, all patients, regardless of group allocation, filled out the Distress Thermometer, and the EuroQoL EQ-5D-5L Health Questionnaire again (post-test measures), completed the Client Satisfaction Questionnaire, CSQ-8, and responded to the questionnaire and the same open-ended questions assessing satisfaction with the treatment received. These questions referred to their general inpatient experience rather than to the intervention itself, allowing comparison between groups while minimizing expectancy effects.

The intervention model developed at the SSPU, grounded in a comprehensive and multidisciplinary approach designed to deliver comprehensive care to hospitalized patients with mental health disorders, consists of the following key components:

1- Psychiatric Intervention: Includes clinical assessment, diagnosis, and the implementation of both pharmacological and non-pharmacological treatment plans tailored to the individual needs of each patient.
2- Individual and Family Psychological Intervention: Clinical psychology provides therapeutic support at both individual and family levels, helping families understand the illness and the unique characteristics of each patient while offering guidance to facilitate adaptation within the family context.
3- Family Interviews: Structured sessions designed to enhance understanding of the therapeutic process, improve communication, and provide tools that foster positive coexistence and support for the patient within their social environment.
4- Nursing Intervention: Encompasses the assessment of overall health status, monitoring of functional patterns, development of individualized care plans, emotional support, supervision of medication adherence, and promotion of autonomy in activities of daily living.
5- Social Work Intervention: Focuses on identifying and addressing social factors that may hinder recovery, with the goal of supporting hospital discharge and ensuring sustained reintegration into the community.
6- Psychoeducation, Emotional Regulation, and Social Skills Workshops: Led by psychologists, nurses, and occupational therapists, these workshops aim to increase illness awareness, develop emotional self-regulation strategies, and strengthen interpersonal and social competencies.
7- Patient Assembly: An open participatory space where patients can share their perspectives, express concerns, and suggest improvements regarding the functioning of the unit, fostering active listening and shared responsibility in the therapeutic process.
8- Occupational and Cognitive Stimulation Workshops: Activities designed to promote cognitive, physical, and social development. Among these is the Expert Patient Workshop, where individuals share their skills or experiences, reinforcing their sense of self-efficacy and personal competence.
9- Constructivist Contribution: Incorporates narrative, expressive, and creative therapies—including visual arts, music, and dance—that facilitate emotional expression, meaning-making, and the reconstruction of personal narratives related to the experience of mental illness.
10- Interdisciplinary Coordination: Regular meetings among professionals ensure the integration and coherence of all interventions, promoting a comprehensive understanding of the patient and a unified therapeutic approach.
11- Social Reintegration Strategies: Gradual measures such as phone calls, visits, and authorized outings are implemented to facilitate the patient’s safe and supported reintegration into daily life.

With regard to the yoga intervention, inpatients participated in structured sessions designed to promote physical wellbeing, relaxation, and mind–body awareness. The sessions aimed to reduce stress and anxiety while supporting emotional stabilization. The intervention was delivered by a psychologist and certified yoga therapist (C-IAYT, International Association of Yoga Therapists) with 10 years of experience applying yoga therapeutically in mental health settings and 20 years of experience as a certified yoga instructor. The yoga sessions were conducted in a room at the 30-bedded SSPU appropriate for yoga practice. Each session, of an hour, was conducted twice weekly. Attendance at the yoga sessions varied, with up to 13 patients per session. Sessions were offered in parallel to routine inpatient care rather than in fixed blocks. Participants assigned to the experimental group took part in the yoga sessions in addition to standard comprehensive care, while participants in the control group received standard comprehensive care only. To minimize the risk of contamination between groups, several measures were implemented. Yoga sessions were scheduled separately from other therapeutic activities, participants were explicitly instructed not to discuss intervention-specific content with other patients, and clinical staff supervised adherence to group allocation during the inpatient stay.

The intervention was developed based on standardized yoga protocols for psychiatric disorders—including schizophrenia, bipolar disorder, addictions, and depression—previously described and validated in studies conducted at the National Institute of Mental Health and Neurosciences (NIMHANS), India (31). In addition, certain practices derived from the Gitananda Rishiculture Asthanga Yoga Tradition teachings were incorporated. Specifically, these included Jattis and Kriyas (warm-up techniques) and Brahma Mudra (32, 33).

The protocol for the specific yoga intervention is detailed in Table 1.

**Table 1:** Protocol for the specific yoga intervention.

Yogic practice	Frequency	Duration
Omkar Chanting	3 times	2 min
Jattis and Kriyas (Warm-up techniques): Shake it out body parts Nada Bhavana Shuddhi Kriya Hakara Kriya	1 round 1 round 1 round	12 min
Brahma Mudra Shavasa Kriya (Twisting) Shavasa Kriya (Hand stretch breathing)Hastottanasana Padahastasana Shvasa KriyaTrikonasana Shvasa Kriya	1 round 10 rounds 3 rounds 10 rounds 10 rounds	15 min
Surya Namaskar	2 rounds	2 min
Sheeghra Shitilikaran Upaya (Quick Relaxation)	1 round	5 min
Vyaghra Shvasa Kriya Shashankasana Shvasa Kriya	10 rounds 10 rounds	4 min
Ardha Ustrasana Vakrasana Ardha Matsyasana Bhujangasana Ardha Shalabasana Dhanurasana	1 round 1 round 1 round 1 round 1 round 1 round	10 min
Pranayama: Bhastrika Bhramari	2 cycles (10 strokes/cycle) 10 rounds	6 min
Nadanusandhana (AA, UU, MM, A-U-M chanting)	3 rounds (each sound)	4 min

### Data analyses

A quantitative analysis was conducted on the results obtained from the standardized questionnaires assessing distress, general health status and patient functionality, and satisfaction with the treatment received during admission and stay. In addition, an exploratory qualitative study was performed based on patients’ responses regarding their recovery and treatment experience in the SSPU, collected through the open-ended questionnaire.

The quantitative analysis was conducted using Jamovi version 2.4.12.

Participants were categorized into two age groups (≤40 and >40 years) and two gender groups (male and female) for stratified randomization and descriptive purposes. First, the mean attendance rate and the frequencies of attendance at the yoga sessions were calculated. Subsequently, variables related to sociodemographic characteristics, lifestyle habits, family psychiatric history, comorbidities, psychiatric diagnosis, and the patient’s self-perceived prior health status were compared between the control and experimental groups, in order to determine whether there were any statistically significant differences between the groups prior to treatment that should be considered in subsequent analyses.

Normality assumptions were assessed prior to statistical analysis. As several variables did not meet criteria for normal distribution, non-parametric statistical tests were applied. Accordingly, within-group pre–post changes were examined using the Wilcoxon signed-rank test, while between-group comparisons (control vs. experimental) were performed using the Mann–Whitney U test. For between-group comparisons using the Mann–Whitney U test, effect sizes were estimated using the rank-biserial correlation (RBC).

Pre–post comparisons were performed to examine overall changes during hospitalization across the full sample, regardless of group allocation. Between-group comparisons were conducted separately using post-intervention values. These analyses were conducted separately in order to appropriately address the two analytical dimensions (time and group) without violating statistical assumptions. Due to the use of non-parametric tests, interaction effects between time and group were not formally tested.

Given the distribution of the data, central tendency is primarily reported using medians, while means and standard deviations are presented for descriptive purposes where appropriate.

To complement the non-parametric analyses, ANCOVA was performed for the main outcome variables using post-intervention scores as dependent variables and baseline values as covariates. Group was included as a fixed factor, and effect sizes were estimated using omega squared (ω^2^).

The Client Satisfaction Questionnaire (CSQ), which assesses satisfaction with the treatment received, was administered only once at discharge. A Mann–Whitney U test for independent samples was then conducted to determine whether there were significant differences in CSQ scores between the control and experimental groups. Therefore, CSQ results represent post-intervention between-group comparisons and do not reflect pre–post changes.

In addition to the quantitative analyses, a descriptive qualitative analysis was conducted on the open-ended responses provided by participants. These qualitative data were collected as part of the same assessment protocol and were analyzed using a thematic approach to complement and contextualize the quantitative results. This qualitative component did not constitute an independent study nor follow a separate sampling strategy.

The exploratory qualitative analysis examined patients’ perceptions of the yoga intervention implemented in the SSPU, providing insight into their subjective experiences and the meaning patterns emerging from their narratives. The analysis combined thematic coding techniques supported by the conversational AI tool in ATLAS.ti^®^ (version 25.0.1; Scientific Software Development GmbH, Germany).

Following Braun and Clarke’s (34) framework, verbatim responses were coded inductively to generate a code tree of codes, subthemes, and themes. ATLAS.ti was used to support data management and coding. AI-assisted tools were employed exclusively to facilitate code organization, while theme identification and interpretation were carried out by the research team, in line with the theoretical principles of thematic analysis. Researchers then manually refined the categories to address redundancies. Co-occurrence analysis was used to identify subthemes appearing together within the same quotations, offering a deeper understanding of how participants conceptualize the yoga intervention. This analysis was conducted using ATLAS.ti to examine relationships between the identified subthemes and to support the interpretation of the qualitative findings.

## Results

The total number of participants in the study was 113 patients. There were 5 null cases and 2 missing cases. Following the criteria established in the study design, 6 cases who attended only one yoga session were excluded. The final number of participants was 100 of both genders (45 females and 55 males) between 18 and 70 years. Of the total sample, 52 participants were assigned to the experimental group, which received the standard comprehensive care supplemented with a specific yoga intervention, and 48 participants were assigned to the control group, which received the standard comprehensive care alone. The mean age of the experimental group was 43.2 ± 12.7 years, and that of the control group was 44.9 ± 15.7 years. The mean attendance rate for the yoga sessions was 84.4% ± 22.3, calculated based on attendance records completed for each session. Table 2 summarizes participants’ attendance and adherence to the intervention sessions. These data are provided to describe feasibility and engagement with the intervention and to contextualize the interpretation of the clinical outcomes.

**Table 2:** Frequencies of attendance at the yoga sessions.

No. of sessions	Frequency	% of total
2	6	11.5%
3	10	19.2%
4	19	36.5%
5	4	7.7%
6	6	11.5%
7	1	1.9%
8	3	5.8%
9	1	1.9%
10	1	1.9%
13	1	1.9%

The results of the chi-square tests (for categorical variables) and Mann–Whitney U tests (for numerical variables) indicated that there were no statistically significant differences between the groups in the variables analyzed, meaning that the groups were comparable, except for the variable “Tobacco Use” (Tables 3, 4).

**Table 3:** Descriptive statistics: frequency and percentage, and chi-square tests for categorical variables.

Variable	CG *n* (%)	EG *n* (%)	*p*
Gender			0.809
Female	21 (46.7)	24 (53.3)	
Male	27 (49.1)	28 (50.9)	
Marital status			0.734
Single	35 (47.3)	39 (52.7)	
Married	7 (58.3)	5 (41.7)	
Separated	2 (66.7)	1 (33.3)	
Divorced	2 (28.6)	5 (71.4)	
Widowed	2 (50.0)	2 (50.0)	
Education			0.442
Primary	5 (45.5)	6 (54.5)	
Lower secondary	12 (36.4)	21 (63.6)	
Upper secondary	9 (50.0)	9 (50.0)	
Vocational training	17 (60.7)	11 (39.3)	
Bachelor’s degree	4 (44.4)	5 (55.6)	
Postgraduate	1 (100)	0	
Occupation			0.191
Unemployed	2 (18.2)	9 (81.8)	
Self-employed	0	1 (100)	
Employee	38 (51.4)	36 (48.6)	
Public servant	1 (33.3)	2 (66.7)	
Retired	6 (75.0)	2 (25.0)	
Student	1 (50.0)	1 (50.0)	
Housework	0	1 (100)	
Family psychiatric history			0.064
No	31 (56.4)	24 (43.6)	
Yes	17 (37.8)	28 (62.2)	
Tobacco use			**0.047**
No	28 (58.3)	20 (41.7)	
Yes	20 (38.5)	32 (61.5)	
Caffeine use			0.397
No	12 (41.4)	17 (58.6)	
Yes	36 (50.7)	35 (49.3)	
Alcohol use			0.812
None	30 (50.0)	30 (50.0)	
Moderate	17 (45.9)	20 (54.1)	
Harmful	1 (33.3)	2 (66.7)	
Drug use			0.166
No	39 (52.0)	36 (48.0)	
Yes	9 (36.0)	16 (64.0)	
Physical exercise			0.327
None	19 (41.3)	27 (58.7)	
Light	19 (50.0)	19 (50.0)	
Moderate	10 (62.5)	6 (37.5)	
Comorbidities			0.959
No	33 (47.8)	36 (52.2)	
Yes	15 (48.4)	16 (51.6)	
Psychiatric diagnosis			0.539
Psychosis	9 (60.0)	6 (40.0)	
Schizophrenia	7 (36.8)	12 (63.2)	
Schizoaffective disorder	3 (42.9)	4 (57.1)	
Bipolar disorder	3 (75.0)	1 (25.0)	
Bipolar mania	5 (55.6)	4 (44.4)	
Personality disorder	2 (50.0)	2 (50.0)	
Delusional disorder	3 (50.0)	3 (50.0)	
Depression	3 (21.4)	11 (78.6)	
Mania	1 (50.0)	1 (50.0)	
Organic affective disorder	1 (100)	0	
Hypochondriac disorder	1 (100)	0	
Psychotic episode	6 (66.7)	3 (33.3)	
Paranoid schizophrenia	3 (60.0)	2 (40.0)	
Toxic psychosis	0	1 (100)	
Paraphrenia	1 (100)	0	
Anxious-depressive disorder	0	1 (100)	
Depressive episode	0	1 (100)	
Self-perceived prior health			0.478
Malaise	30 (45.5)	36 (54.5)	
Wellbeing	18 (52.9)	16 (47.1)	

CG, Control group; EG, Experimental Group; n, Frequency; %, Percentage.

Bold values indicate statistically significant results (*p* < 0.05).

**Table 4:** Descriptive statistics: frequency, mean and standard deviation, and Mann–Whitney U tests for numerical variables.

Variable	GC (n)	GC M(SD)	GE (n)	EG M(SD)	*p*
Age (years)	48	44.9(15.7)	52	43.2(12.7)	0.512
Length of stay	48	21(2.5)	52	20.3(1.5)	0.197
Restraints	3	5(7)	5	1.6(0.9)	0.878

CG, Control group; EG, Experimental Group; n, Frequency; M, Mean; SD, Standard Deviation.

There were no significant differences between the groups regarding the length of stay and the number of physical restraints. The mean length of stay for the control group was 21 ± 2.5 days, and for the experimental group, 20.3 ± 1.5 days. The mean number of physical restraints was 5 ± 7 in 3 participants of the control group, and 1.6 ± 0.9 in 5 participants of the experimental group.

Using a Mann–Whitney U test for independent samples, no significant differences were found in the pre-treatment measurements of the dependent variables between the groups, indicating that the groups were homogeneous at baseline (Table 5). Effect sizes for these between-group comparisons were small, as indicated by RBC values. Although post-intervention scores for emotional distress were slightly lower, and scores for health status and functional capacity were slightly higher in the experimental group compared to the control group, the Mann–Whitney U test showed no statistically significant differences between the groups after the intervention. ANCOVA analyses were conducted to adjust post-intervention outcomes for baseline values. Baseline scores were significant covariates for most variables. After adjustment, no significant group effects were observed for most outcomes; however, a significant group effect was found for the EQ-5D self-care dimension (Mann–Whitney U *p* = 0.02; ANCOVA *p* = 0.017), with a small effect size.

**Table 5:** Descriptive statistics and between-group comparisons of post-intervention outcomes, including Mann–Whitney U test and ANCOVA adjusted for baseline values.

Variable	Group	Mean	Median	SD	*p*-value Mann Whitney U test	*p*-value ANCOVA	Effect size (ω^2^)
DT_PRE	Control	6.60	7.50	3.43	0.41	0.59	0.007
	Experimental	6.96	8.50	3.44			
DT_POST	Control	2.65	1.00	3.34			
	Experimental	2.35	1.00	2.77			
EQ_M_PRE	Control	0.40	0.00	0.71	0.26	0.26	0.021
	Experimental	0.34	0.00	0.68			
EQ_M_POST	Control	0.27	0.00	0.49			
	Experimental	0.17	0.00	0.43			
EQ_SC_PRE	Control	0.38	0.00	0.82	**0.02**	**0.017**	**0.042**
	Experimental	0.42	0.00	0.83			
EQ_SC_POST	Control	0.23	0.00	0.63			
	Experimental	0.04	0.00	0.19			
EQ_UA_PRE	Control	0.73	0.00	1.09	0.36	0.35	0.008
	Experimental	0.98	0.00	1.20			
EQ_UA_POST	Control	0.44	0.00	0.87			
	Experimental	0.35	0.00	0.71			
EQ_PD_PRE	Control	1.06	1.00	1.12	0.67	0.68	0.006
	Experimental	1.21	1.00	1.16			
EQ_PD_POST	Control	0.58	0.00	0.82			
	Experimental	0.52	0.00	0.67			
EQ_AD_PRE	Control	1.60	2.00	1.27	0.44	0.45	0.005
	Experimental	2.04	2.00	1.50			
EQ_AD_POST	Control	0.67	0.00	1.06			
	Experimental	0.90	1.00	0.99			
EQ_TOT_PRE	Control	4.17	3.50	3.45	0.31	0.31	0.014
	Experimental	5.00	5.00	3.67			
EQ_TOT_POST	Control	2.19	1.00	2.94			
	Experimental	1.98	1.00	2.14			
VAS_PRE	Control	68.80	80.00	28.32	0.45	0.45	0.006
	Experimental	59.71	67.50	26.48			
VAS_POST	Control	74.27	80.00	22.05			
	Experimental	75.29	80.00	20.30			

DT_PRE, Distress Thermometer Pre-test; DT_POST, Distress Thermometer Post-Test; EQ_M_PRE, EuroQoL Mobility Pre-Test; EQ_M_POST, EuroQoL Mobility Post-Test; EQ_SC_PRE, EuroQoL Self-care Pre-Test; EQ_SC_POST, EuroQoL Self-care Post-Test; EQ_UA_PRE, EuroQoL Usual Activities Pre-Test; EQ_UA_POST, EuroQoL Usual Activities Post-Test; EQ_PD_PRE, EuroQoL Pain/discomfort Pre-Test; EQ_PD_POST, EuroQoL Pain/discomfort Post-Test; EQ_AD_PRE, EuroQoL Anxiety/Depression Pre-Test; EQ_AD_POST, EuroQoL Anxiety/Depression Post-Test; EQ_TOT_PRE; EuroQoL Total score Pre-test; EQ_TOT_POST, EuroQoL Total score Post-test; VAS_PRE, EuroQoL Visual Scale Pre-test; VAS_POST, EuroQoL Visual Scale Post-test; SD, Standard Deviation; ω^2^, Omega squared.

Bold values indicate statistically significant results (*p* < 0.05).

Therefore, the first and second hypotheses, which proposed a statistically significant reduction in emotional distress and a statistically significant improvement in overall health status and functional capacity in the experimental group compared to the control group following the intervention, were not supported by the results, as no statistically significant differences were observed between groups, except for the self-care dimension, in which the experimental group showed a significant post-intervention improvement compared to the control group.

The scores of the Client Satisfaction Questionnaire (CSQ) indicate that satisfaction with the treatment received during admission and stay in the SSPU was very high in both groups (Table 6). The mean item scores ranged from 3.17 to 3.52 in the control group and from 3.29 to 3.71 in the experimental group, with a maximum possible score of 4 points per item. The mean total scores were 27.23 for the control group and 28.56 for the experimental group, with a maximum possible score of 32 points. The Mann–Whitney U test revealed statistically significant differences in the total CSQ score (*p* = 0.045; RBC = 0.231) and in items CSQ_5 (*p* = 0.034; RBC = 0.211) and CSQ_6 (*p* = 0.041; RBC = 0.208), with the experimental group reporting higher overall satisfaction (Table 7). These findings support the third hypothesis, indicating that while all patients reported high satisfaction with the treatment, those in the experimental group perceived their experience in the SSPU significantly more positive than the control group. In addition, the experimental group reported greater satisfaction with the amount of help received and had a more positive perception of the support provided to help them better cope with their problems.

**Table 6:** Descriptive statistics of Client Satisfaction Questionnaire (CSQ) Scores for the control and experimental groups.

Variable	Group	Mean	Median	SD
CSQ_1	Control	3.35	3.00	0.57
	Experimental	3.38	3.00	0.66
CSQ_2	Control	3.48	4.00	0.65
	Experimental	3.60	4.00	0.60
CSQ_3	Control	3.17	3.00	0.88
	Experimental	3.29	3.50	0.80
CSQ_4	Control	3.50	4.00	0.77
	Experimental	3.71	4.00	0.61
CSQ_5	Control	3.42	3.50	0.68
	Experimental	3.67	4.00	0.55
CSQ_6	Control	3.35	3.00	0.73
	Experimental	3.63	4.00	0.53
CSQ_7	Control	3.44	3.50	0.65
	Experimental	3.62	4.00	0.60
CSQ_8	Control	3.52	4.00	0.85
	Experimental	3.63	4.00	0.74
CSQ_TOT	Control	27.23	28.00	3.99
	Experimental	28.56	30.00	3.90

CSQ_1, Item 1; CSQ_2, Item 2; CSQ_3, Item 3; CSQ_4, Item 4; CSQ_5, Item 5; CSQ_6, Item 6; CSQ_7, Item 7; CSQ_8, Item 8; CSQ_TOT, Total score; SD, Standard Deviation.

**Table 7:** Differences between the control and experimental groups on Client Satisfaction Questionnaire (CSQ) scores, using Mann–Whitney U test.

Variable	*U*	*p*	Rank biserial correlation
CSQ_1	1,188	0.643	0.049
CSQ_2	1,122	0.313	0.101
CSQ_3	1,165	0.537	0.067
CSQ_4	1,062	0.111	0.149
CSQ_5	985	**0.034**	0.211
CSQ_6	989	**0.041**	0.208
CSQ_7	1,050	0.114	0.159
CSQ_8	1,167	0.478	0.065
CSQ_TOT	960	**0.045**	0.231

CSQ_1, Item 1; CSQ_2, Item 2; CSQ_3, Item 3; CSQ_4, Item 4; CSQ_5, Item 5; CSQ_6, Item 6; CSQ_7, Item 7; CSQ_8, Item 8; CSQ_TOT, Total score.

Bold values indicate statistically significant results (*p* < 0.05).

Finally, the Wilcoxon Signed-Rank Test for paired samples revealed statistically significant pre- to post-test improvements across all dependent variables for both groups (*p* < 0.05), including reductions in perceived distress and health and functional problems, as well as enhanced overall self-rated health (Tables 8, 9). Effect size estimates based on rank-biserial correlation indicated large effects for emotional distress (RBC = 0.859), overall health status (RBC = 0.517), self-care (RBC = 0.723), usual activities (RBC = 0.664), pain/discomfort (RBC = 0.684), anxiety/depression (RBC = 0.768), and overall EQ-5D scores (RBC = 0.770), while a moderate effect was observed for the visual analogue scale (VAS; RBC = 0.440). These results confirm the fourth hypothesis, showing that upon discharge from the SSPU, patients exhibited statistically significant decreases in emotional distress and improvements in health and functional outcomes, regardless of group assignment.

**Table 8:** Descriptive statistics for the pre- and post-test measurements of the dependent variables for all patients.

Variable	Mean	Median	SD
DT_PRE	6.79	8.00	3.42
DT_POST	2.49	1.00	3.05
EQ_M_PRE	0.37	0.00	0.69
EQ_M_POST	0.22	0.00	0.46
EQ_SC_PRE	0.40	0.00	0.82
EQ_SC_POST	0.13	0.00	0.46
EQ_UA_PRE	0.86	0.00	1.15
EQ_UA_POST	0.39	0.00	0.79
EQ_PD_PRE	1.14	1.00	1.14
EQ_PD_POST	0.55	0.00	0.74
EQ_AD_PRE	1.83	2.00	1.40
EQ_AD_POST	0.79	0.00	1.03
EQ_TOT_PRE	4.60	4.00	3.57
EQ_TOT_POST	2.08	1.00	2.54
VAS_PRE	64.07	70.00	27.62
VAS_POST	74.80	80.00	21.06

DT_PRE, Distress Thermometer Pre-test; DT_POST, Distress Thermometer Post-Test; EQ_M_PRE, EuroQoL Mobility Pre-Test; EQ_M_POST, EuroQoL Mobility Post-Test; EQ_SC_PRE, EuroQoL Self-care Pre-Test; EQ_SC_POST, EuroQoL Self-care Post-Test; EQ_UA_PRE, EuroQoL Usual Activities Pre-Test; EQ_UA_POST, EuroQoL Usual Activities Post-Test; EQ_PD_PRE, EuroQoL Pain/discomfort Pre-Test; EQ_PD_POST, EuroQoL Pain/discomfort Post-Test; EQ_AD_PRE, EuroQoL Anxiety/Depression Pre-Test; EQ_AD_POST, EuroQoL Anxiety/Depression Post-Test; EQ_TOT_PRE; EuroQoL Total score Pre-test; EQ_TOT_POST, EuroQoL Total score Post-test; VAS_PRE, EuroQoL Visual Scale Pre-test; VAS_POST, EuroQoL Visual Scale Post-test; SD, Standard Deviation.

**Table 9:** Differences between pre- and post-test measurements of the dependent variables for all patients, using Wilcoxon Signed-Rank Test.

Variable	*W*	*p*	Rank biserial correlation
DT_PRE – DT_POST	3891	**< 0.001**	0.859
EQ_M_PRE – EQ_M_POST	247	**0.015**	0.517
EQ_SC_PRE – EQ_SC_POST	218	**0.003**	0.723
EQ_UA_PRE – EQ_UA_POST	717	**< 0.001**	0.664
EQ_PD_PRE – EQ_PD_POST	1441	**< 0.001**	0.684
EQ_AD_PRE – EQ_AD_POST	1954	**< 0.001**	0.768
EQ_TOT_PRE – EQ_TOT_POST	3235	**< 0.001**	0.770
VAS_PRE – VAS_POST	999	**< 0.001**	0.440

DT_PRE, Distress Thermometer Pre-test; DT_POST, Distress Thermometer Post-Test EQ_M_PRE, EuroQoL Mobility Pre-Test; EQ_M_POST, EuroQoL Mobility Post-Test; EQ_SC_PRE, EuroQoL Self-care Pre-Test; EQ_SC_POST, EuroQoL Self-care Post-Test; EQ_UA_PRE, EuroQoL Usual Activities Pre-Test; EQ_UA_POST, EuroQoL Usual Activities Post-Test; EQ_PD_PRE, EuroQoL Pain/discomfort Pre-Test; EQ_PD_POST, EuroQoL Pain/discomfort Post-Test; EQ_AD_PRE, EuroQoL Anxiety/Depression Pre-Test; EQ_AD_POST, EuroQoL Anxiety/Depression Post-Test; EQ_TOT_PRE; EuroQoL Total score Pre-test; EQ_TOT_POST, EuroQoL Total score Post-test; VAS_PRE, EuroQoL Visual Scale Pre-test; VAS_POST, EuroQoL Visual Scale Post-test.

Bold values indicate statistically significant results (*p* < 0.05).

Regarding the exploratory qualitative analysis, a total of 20 codes were identified, which were organized into six subthemes and grouped under three overarching themes, as presented in Table 10. All participants included in the study (*n* = 100) completed the open-ended questions.

**Table 10:** Themes, subthemes, and representative verbatims identified in the exploratory qualitative analysis.

Theme	Subtheme	Representative verbatim
Benefits of yoga practice	Relaxation	“It helps me relax. It gives me peace. I disconnect… I forget about everything.”
	Emotional regulation	“It helps me identify and express my emotions. It helps me manage anger.”
Activities supporting recovery	Yoga as a therapeutic tool	“Yoga helps me channel my energy and reduce anxiety.”
	Complementary activities	“The routine, music, workshops, and interacting with others also help me.”
General perception and subjective experience	Feelings of peace and safety	“I feel more confident. It helps me manage my emotions.”
	Improved communication and empathy	“Yoga helps me communicate better and feel more empathy.”

Theme 1: benefits of yoga practice.

Patients expressed highly positive evaluations of the intervention, emphasizing tranquility and emotional wellbeing.

Subtheme 1.1: relaxation: yoga practice was experienced as a space for calm, disconnection, and emotional regulation.

*“It helps me relax. It gives me peace. I disconnect… I forget about everything.”*

Subtheme 1.2: emotional regulation: many patients reported that yoga practice allowed them to identify difficult or suppressed emotions and express states such as anger more adaptively.

*“It helps me identify and express my emotions.,” “It helps me manage anger.”*

Theme 2: activities supporting recovery.

In addition to yoga, patients mentioned other activities significant to their therapeutic process.

Subtheme 2.1: yoga as a therapeutic tool: yoga practice was perceived as a key tool for channeling energy, emotional regulation, and stress management.

*“Yoga helps channel energy and reduces anxiety.,” “It helps me in my daily routine.”*

Subtheme 2.2: complementary activities: patients highlighted music therapy, workshops, outings, and social interaction as essential elements for recovery.

Theme 3: general perception and subjective experience.

Patients described the intervention as a transformative experience that enhanced emotional wellbeing and personal sense of safety.

Subtheme 3.1: feelings of peace and safety: yoga practice was experienced as a space that reduced internal tension and promoted self-confidence.

*“I feel more confident.,” “It helps me manage my emotions.”*

Subtheme 3.2: improved communication and empathy: patients perceived that group activities, including yoga, improved communication among peers and strengthened relationships with healthcare staff.

*“Yoga helps me communicate better and feel more empathy.”*

### Co-occurrences

1. Relaxation ↔ Emotional Regulation: This co-occurrence indicated that patients did not clearly distinguish between relaxing and regulating emotions. For them, relaxation was equivalent to regulation, as the bodily experience of calming down had direct effects on how they managed difficult emotions. Yoga practice was experienced as a space producing a dual effect: tension reduction and enhanced emotional control.
2. Improved communication and empathy ↔ Emotional Support: Patients associated yoga practice with a supportive environment, as they felt accompanied, shared experiences, and felt understood. The intervention also influenced relational dimensions, reduced isolation, improved self-esteem, and strengthened the internal support network within the group.
3. Feelings of peace and safety ↔ Self-Awareness: Patients reported that practicing yoga improved their feelings of peace and safety, enhanced understanding of their emotions, and fostered greater self-confidence and internal clarity. This co-occurrence suggested that yoga practice promoted personal growth processes, as bodily work facilitated emotional and cognitive introspection.

## Discussion

The most significant finding of this study was the beneficial effect of the comprehensive mental health intervention on patients’ wellbeing during their stay in the Short-Stay Psychiatric Unit (SSPU). In addition, participants who received a specific yoga intervention as part of the program reported higher satisfaction with their treatment.

Quantitative analyses showed that all participants experienced a significant decrease in perceived distress and in health and functional problems, along with a significant improvement in overall self-rated health following the comprehensive intervention (Table 9). Among participants in the yoga intervention, the self-care dimension showed a significant post-intervention improvement compared to the control group (Table 5). Nevertheless, these participants reported higher overall satisfaction, particularly regarding the perceived amount of help received and the extent to which the services helped them manage their problems more effectively (Table 7). Both non-parametric between-group comparisons and ANCOVA-adjusted analyses were conducted to provide a robust assessment of group differences. While ANCOVA did not reveal additional significant effects beyond those identified with non-parametric tests, the convergence of findings across both analytical approaches supports the robustness and consistency of the results. These findings are consistent with previous research indicating that psychiatric patients engaging in yoga interventions often report more positive perceptions of treatment, even when measurable clinical improvements in anxiety or depression symptoms are limited (22, 23). Similarly, studies integrating yoga as a complementary approach within residential and partial hospitalization programs have highlighted its perceived benefits for emotional regulation, coping, and the overall treatment experience (24, 35).

Although the yoga intervention did not produce statistically significant effects on emotional distress or overall health beyond the comprehensive program, qualitative findings provided important complementary insight into patients’ experiences. Participants consistently described the yoga sessions as calming and supportive for emotional regulation, as well as a meaningful space for channeling emotions, managing stress, and enhancing self-confidence, personal safety, and overall emotional wellbeing. In addition, yoga, together with other therapeutic activities such as music therapy, thematic workshops, community outings, and opportunities for social engagement, was perceived as an integral component of recovery. Group-based activities, including yoga, facilitated peer communication and fostered positive, collaborative interactions with healthcare staff.

To our knowledge, few studies have explored patients’ experiences of yoga in inpatient psychiatric settings using qualitative approaches. Existing research has identified themes such as emotional regulation, self-awareness, and improved coping (36–38), but none have conducted comprehensive thematic analyses in acute or short-stay units. The present study thus provides novel qualitative evidence of patients’ experiences with yoga in this intensive inpatient context. These qualitative insights, particularly regarding relaxation, emotional processing, and perceived safety, overlap conceptually with themes reported in the study by Marsh et al. (35). Co-occurrence analysis further suggests that experiences such as relaxation, emotional regulation, and body awareness are closely interrelated, reflecting experiential patterns rather than distinct therapeutic mechanisms.

Participants perceived yoga sessions as supportive and emotionally meaningful within the broader context of inpatient care, without implying causal or clinical effects beyond those captured in the quantitative analyses. Overall, these observations indicate that yoga sessions are highly acceptable and feasible in acute psychiatric care. This is consistent with reports on yoga-based interventions in mental health contexts, which have documented high levels of engagement and acceptability (39). While participants valued and engaged positively with the intervention, these findings primarily reflect acceptability and patient experience rather than clinical effectiveness, which was addressed separately in the quantitative analyses.

### Strengths and limitations

Although a wide range of psychosocial and educational interventions are employed in acute and recovery-oriented mental health settings, the present study focused specifically on yoga-based interventions due to their growing use as complementary mind–body approaches. The findings should therefore be interpreted within this specific context and not as a comparison with other therapeutic modalities.

The literature on yoga-based interventions in short-stay psychiatric units remains extremely limited. The present study contributes to addressing this gap by providing empirical data that extend current knowledge regarding the feasibility, implementation, and potential effects of such interventions in these acute clinical settings.

Data collection prioritized patient comfort and feasibility. While a broad range of thematic information was obtained, participants were not required to engage in lengthy interviews. Instead, brief, open-ended questions were integrated into routine therapeutic activities, allowing patients to respond according to their capacity and clinical state. The richness of the qualitative material emerged from aggregating responses across participants, rather than from prolonged individual interviews, thereby minimizing burden in an acute mental health context.

Several limitations should be considered when interpreting the findings. First, no *a priori* sample size calculation was performed, as recruitment depended on the number of patients admitted during the study period. Consequently, the study may have been underpowered to detect small between-group effects, and results should be interpreted cautiously. Second, the use of a non-probabilistic recruitment strategy may limit the generalizability beyond similar inpatient settings. However, random allocation to study groups strengthens internal validity and supports interpretation of group differences as intervention-related. Third, the reliance on self-report measures represents a potential limitation. While these measures were appropriate for capturing patients’ subjective experiences of wellbeing and satisfaction, challenges inherent to the clinical context must be acknowledged. Participants’ mental states upon admission, often marked by confusion, impaired reality testing, and communication difficulties, may have affected the accuracy of pre-test responses. Nevertheless, despite efforts to minimize comprehension difficulties through the use of brief and clinically appropriate instruments, such as the Distress Thermometer and the Visual Analog Scale (VAS) to assess overall health, some inaccuracies may have occurred. For instance, 34 of the 100 participants reported a prior health status of “wellbeing,” which may not fully correspond to their actual clinical presentation. Future research should complement self-report data with clinician-administered assessments, such as symptom scales, functional evaluations, and systematic observation, to enhance measurement accuracy while retaining subjective perspectives. Fourth, the study was conducted within a single SSPU, which may further limit external validity. Although contamination between groups was minimized through separate scheduling of sessions, participant instructions, and staff supervision, the possibility of residual contamination cannot be entirely excluded.

## Conclusion

The comprehensive and multidisciplinary approach implemented in the Short-Stay Psychiatric Unit (SSPU) was associated with high levels of patient engagement and acceptability. However, quantitative analyses showed that, after adjustment for baseline values, no significant differences were observed between groups for most clinical outcomes when a yoga intervention was included, except for a small effect in the EQ-5D self-care dimension. Additionally, patients who participated in the yoga intervention reported significantly greater satisfaction with their treatment.

The qualitative findings provided complementary insight into patients’ experiences, highlighting emotional expression, perceived emotional support, and feelings of peace and safety during hospitalization. These experiences should be interpreted as subjective perceptions rather than as indicators of measurable clinical improvement. In this context, the integration of yoga into routine care appears to be a feasible and well-accepted complementary activity within acute psychiatric settings. While patients perceived the intervention as meaningful and supportive during hospitalization, the present findings suggest that its added clinical benefit beyond standard care is limited.

In summary, this study demonstrates that integrating a yoga-based intervention within a comprehensive mental health program in an acute inpatient setting is feasible, well-received, and perceived as supportive by patients. The results provide novel qualitative evidence of patients’ experiences with yoga in this context, and highlight its potential as a complementary therapeutic activity. Future studies with more controlled designs are needed to clarify its potential clinical and therapeutic benefits.

## Data Availability

The raw data supporting the conclusions of this article will be made available by the authors, without undue reservation.
